# Intramembranous ossification and endochondral ossification are impaired differently between glucocorticoid-induced osteoporosis and estrogen deficiency-induced osteoporosis

**DOI:** 10.1038/s41598-018-22095-1

**Published:** 2018-03-01

**Authors:** Hongyang Zhang, Xiaojuan Shi, Long Wang, Xiaojie Li, Chao Zheng, Bo Gao, Xiaolong Xu, Xisheng Lin, Jinpeng Wang, Yangjing Lin, Jun Shi, Qiang Huang, Zhuojing Luo, Liu Yang

**Affiliations:** 1 Institute of Orthopedic Surgery, Xijing Hospital, The Fourth Military Medical University, Xi’an, 710032 People’s Republic of China; 2 Department of Orthopaedics, Chinese PLA General Hospital, Beijing, 100853 People’s Republic of China; 3 Department of Orthopeadics, Air Force General Hospital, Beijing, 100142 People’s Republic of China; 4 Department of Orthopaedics, First Affiliated Hospital, Chengdu Medical College, No.278, Baoguang Road, Chengdu, SiChuan Province 610500 People’s Republic of China; 5 Department of Orthopedics, No.371 Central Hospital of PLA, Xinxiang, Henan 453000 People’s Republic of China; 6 Lanzhou General hospital of Lanzhou Military Command, Lanzhou, 730050 Gansu People’s Republic of China

## Abstract

A fracture is the most dangerous complication of osteoporosis in patients because the associated disability and mortality rates are high. Osteoporosis impairs fracture healing and prognosis, but how intramembranous ossification (IO) or endochondral ossification (EO) during fracture healing are affected and whether these two kinds of ossification are different between glucocorticoid-induced osteoporosis (GIOP) and estrogen deficiency-induced osteoporosis (EDOP) are poorly understood. In this study, we established two bone repair models that exhibited repair via IO or EO and compared the pathological progress of each under GIOP and EDOP. In the cortical drill-hole model, which is repaired through IO, osteogenic differentiation was more seriously impaired in EDOP at the early stage than in GIOP. In the periosteum scratch model, in which EO is replicated, chondrocyte hypertrophy progression was delayed in both GIOP and EDOP. The *in vitro* results were consistent with the *in vivo* results. Our study is the first to establish bone repair models in which IO and EO occur separately, and the results strongly describe the differences in bone repair between GIOP and EDOP.

## Introduction

Osteoporosis is a skeletal disorder characterized by systemically decreased bone mass and bone microarchitecture destruction, with an increased risk of fracture^1^. Fracture is the most dangerous complication of osteoporosis because of the high disability rate and because fractures can raise the mortality rate^2^. The annual costs of osteoporotic fracture treatment are high^3–6^. Current research has mostly focused on the effects of antiresorptive agents^7–10^ and anabolic agents^11,12^ on osteoporotic fracture healing; however, few studies have addressed the mechanisms of osteoporotic fracture healing. To identify an effective approach for osteoporotic fracture treatment, a thorough understanding of the healing mechanism of osteoporotic fractures is important. In the past few decades, researchers have accomplished a great deal to ensure that osteoporosis impairs fracture healing. The results from many studies have verified that fracture healing is delayed during osteoporosis progression^13–15^. However, a few unresolved questions remain.

Fracture healing is a specialized postnatal repair process that recapitulates embryological skeletal development^16^. This healing process generally comprises two forms of bone regeneration: intramembranous ossification and endochondral ossification. However, the form of bone regeneration that is mainly impaired during osteoporotic conditions (primary or secondary) remains unknown. A comprehensive understanding of *in vivo* ossification and defect repair in osteoporosis models will enable explorations of more specific and efficient treatment strategies.

In the present study, we established two injury models that exhibited healing via intramembranous ossification or endochondral ossification and investigated the differences between glucocorticoid-induced osteoporosis (GIOP) and estrogen deficiency-induced osteoporosis (EDOP). The intramembranous ossification model was established by drill-hole injury at the anteriomedialis of the tibia, and the endochondral ossification model was established by periosteum injury at the anteriomedialis of the tibia. Micro-CT was employed to ensure that osteoporosis had been established, decalcified histology was used to describe the callus histopathology features at the tissue level, and immunohistochemistry was performed to evaluate biochemical markers at the protein level. The proliferation ability and differentiation potential of bone marrow stem cells (BMSCs) from both osteoporosis models were also evaluated and compared *in vitro*, enabling our understanding of the *in vivo* repair process. The *in vivo* and *in vitro* data showed that both endochondral ossification and intramembranous ossification were impaired under osteoporotic conditions. However, intramembranous ossification was less affected in GIOP than in EDOP, and no obvious difference in endochondral ossification was apparent between GIOP and EDOP.

## Results

### The establishment of the GIOP and EDOP osteoporosis models

Eight weeks after ovariectomies and methylprednisolone injections in the EDOP and GIOP mice, respectively, distal femurs were scanned to evaluate the osteoporosis models and control groups. The Micro-CT 3D images showed that the trabecular bones in groups GIOP and EDOP were smaller (bone mass was lower), thinner (bone thickness was thinner), and more disorganized (bone structure was disorganized) than those of the group control (CON) (Supplemental Fig. 1A). The bone mineral density (BMD), relative bone volume over total volume (BV/TV), and trabecular number (Tb.N) in the distal femur were significantly lower in groups GIOP and EDOP than in group CON (Supplemental Fig. 1B–D). The trabecular separation (Tb.Sp) was significantly higher in groups GIOP and EDOP than in group CON (Supplemental Fig. 1E). No significant differences in BMD, BV/TV, or Tb.N were evident between the GIOP and EDOP mice, while the Tb.Sp was slightly higher in the EDOP mice than in the GIOP mice. These results indicate that the GIOP model and ovariectomy-induced osteoporosis model were established successfully. The EDOP and GIOP models showed similar bone masses at 8 weeks after ovariectomy and methylprednisolone injections, respectively.

### Intramembranous ossification was impaired more seriously in EDOP than in GIOP at the early stage of bone repair

By drilling a hole on the anteriomedialis of the tibia, we dissected the intramembranous ossification, which was involved in this healing model^17,18^. Using this model, we found that intramembranous ossification in bone repair was impaired under both osteoporotic conditions.

**Figure 1:**
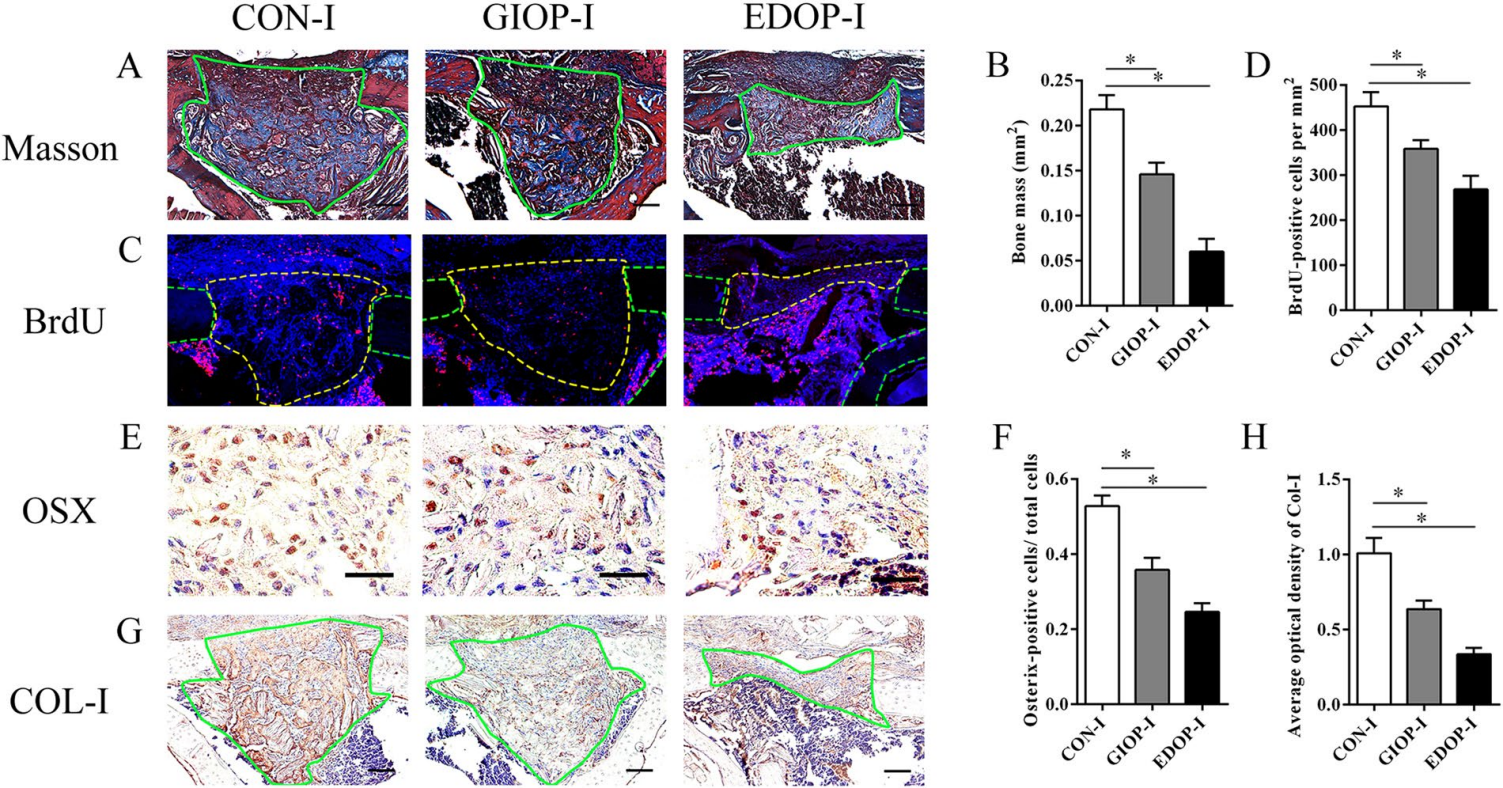
Histological and immunohistochemical analyses of the injury sites 7 days after the drill-hole injury. (**A**) Callus sections stained with Masson’s trichrome. Scale bar, 100 µm. (**B**) Quantitative analyses of blue-stained bone mass, encircled by green lines. (**C**) Immunofluorescence stain of BrdU: positive cells are indicated in red. Each callus is encircled by yellow lines, and cortical bones are encircled by green lines. (**D**) Analyses of the number of BrdU-positive cells. (**E**) Immunohistochemical stain of Osterix: positive cells are indicated in brown. Scale bar, 30 µm. (**F**) Analyses of the number of Osterix-positive cells. (**G**) Immunohistochemical stain of type I collagen (COL-I): COL-I is indicated in brown. Scale bar, 100 µm. (**H**) Analyses of COL-I expression. *P < 0.05.

Seven days after the drill-hole surgery, newly formed woven bone filled in the defect region and most of the medullary cavity in group CON-I. Group GIOP-I also exhibited newly formed woven bone in the defect region and some of the medullary cavity. In group EDOP-I, no woven bone was evident in the medullary cavity, and only a small amount of woven bone was present between the cortical defect. The newly formed bone mass was significantly smaller in group GIOP-I and group EDOP-I than in group CON-I (p < 0.05). The new bone mass was also significantly smaller in group EDOP-I than in group GIOP-I (p < 0.05) (Fig. 1A,B). Cell proliferation is important in the expansion of recruiting progenitors during tissue repair^19^. Immunofluorescence staining suggested that in the callus area, more BrdU-positive cells per callus area were present in group GIOP-I than in group EDOP-I (p < 0.05) (Fig. 1C,D), while the rates was lower in both groups than in group CON-I, indicating impaired cell proliferation in group GIOP-I; however, group EDOP-I displayed a more striking reduction in cell proliferation at the injury location than group CON-I and group GIOP-I. The osteoblast-specific transcription factor Osterix, which acts downstream of Runx2, plays an essential role in osteoblast differentiation^20,21^. Osterix immunohistochemistry suggested that more Osterix-positive cell per callus area were present in group CON-I than in group GIOP-I and group EDOP-I, and the positive cell ratio was also lower in group EDOP-I than in group GIOP-I (p < 0.05) (Fig. 1E,F). Collagen I (COL-I) immunohistochemistry showed similar results indicating that the expression of COL-I was stronger in group GIOP-I than in EDOP-I, while COL-I expression was weaker in both groups than in group CON-I (Fig. 1G,H), suggesting that the early-stage repair process and new bone formation via intramembranous ossification was much more affected after ovariectomy.

**Figure 2:**
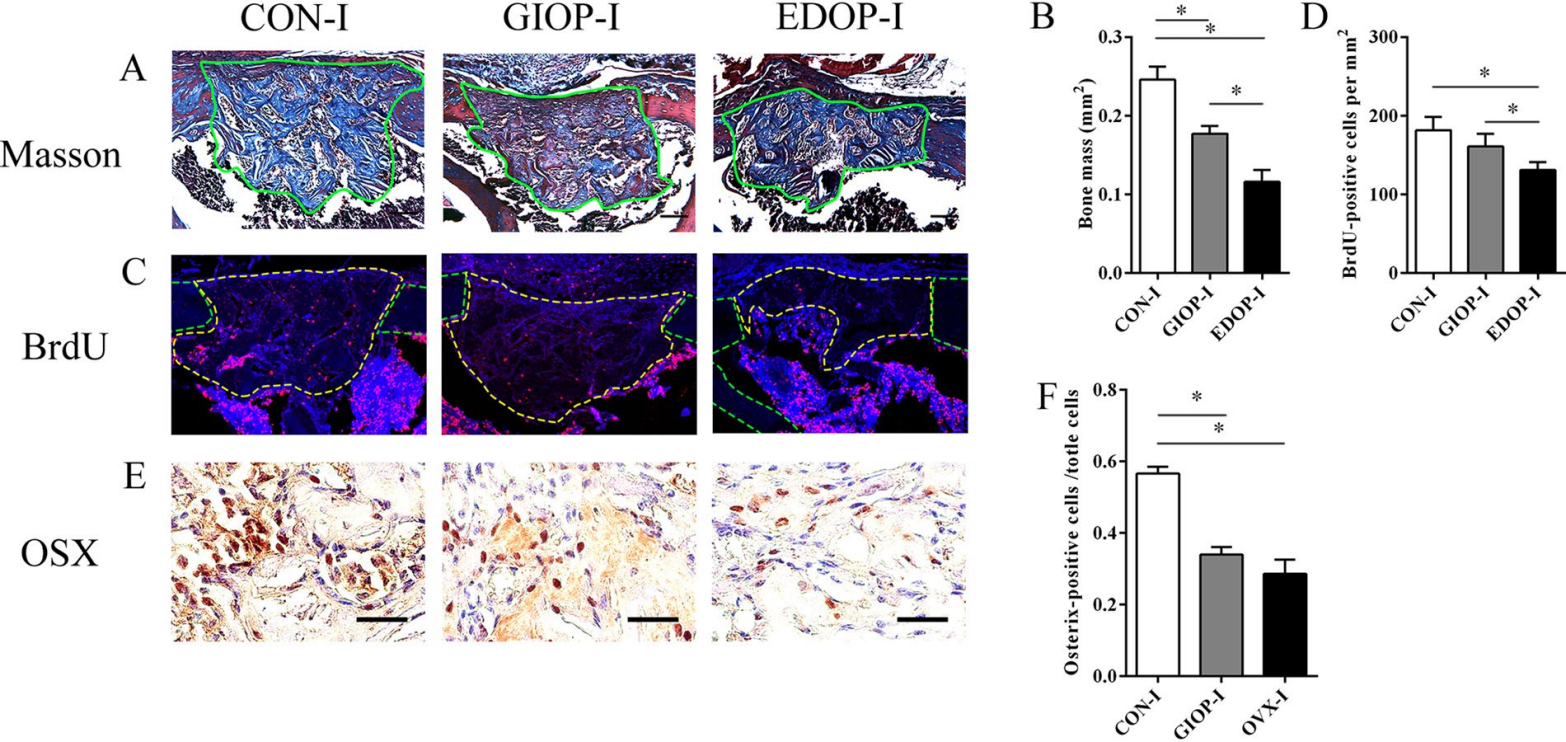
Histological and immunohistochemical analysis of the injury site 10 days after the drill-hole injury. (**A**) Callus sections stained with Masson’s trichrome. Scale bar, 100 µm. (**B**) Quantitative analyses of blue-stained bone mass, encircled by green lines. (**C**) Immunofluorescence stain of BrdU: positive cells are indicated in red. Each callus is encircled by yellow lines, and cortical bones are encircled by green lines. (**D**) Analyses of the number of BrdU-positive cells. (**E**) Immunohistochemical stain of Osterix: positive cells are indicated in brown. Scale bar, 30 µm. (**F**) Analyses of the number of Osterix-positive cells (right). *P < 0.05.

Ten days after surgery, the newly formed woven bone became spicules bone in group CON-I. In group GIOP-I, the callus still comprised newly formed woven bone, while in group EDOP-I, newly formed woven bone connected the cortical gap, but the amount was still low in the medullary (Fig. 2A). The newly formed bone mass slightly increased in group CON-I and group GIOP-I and increased dramatically in group EDOP-I because the callus of these mice were too small on day 7 (Figs 1B and 2B). After statistically analyzing the newly formed bone mass, the callus was still larger in group CON-I than in both group GIOP-I and group EDOP-I. In addition, consistent with the data from day 7, the callus was larger in group GIOP-I than in group EDOP-I (Fig. 2B). BrdU immunofluorescence staining showed that the rate of positive cells in the callus area was much higher in group CON-I than in both osteoporotic models (Fig. 2C,D). Osterix staining suggested that the ratios of Osx+ osteoprogenitor cells in the callus were similar between group CON-I and group GIOP-I, while group EDOP-I displayed far fewer Osx+ cells at the injury site (Fig. 2E,F).

### Bone remodeling in the cortical drill-hole model was also delayed in GIOP and EDOP

**Figure 3:**
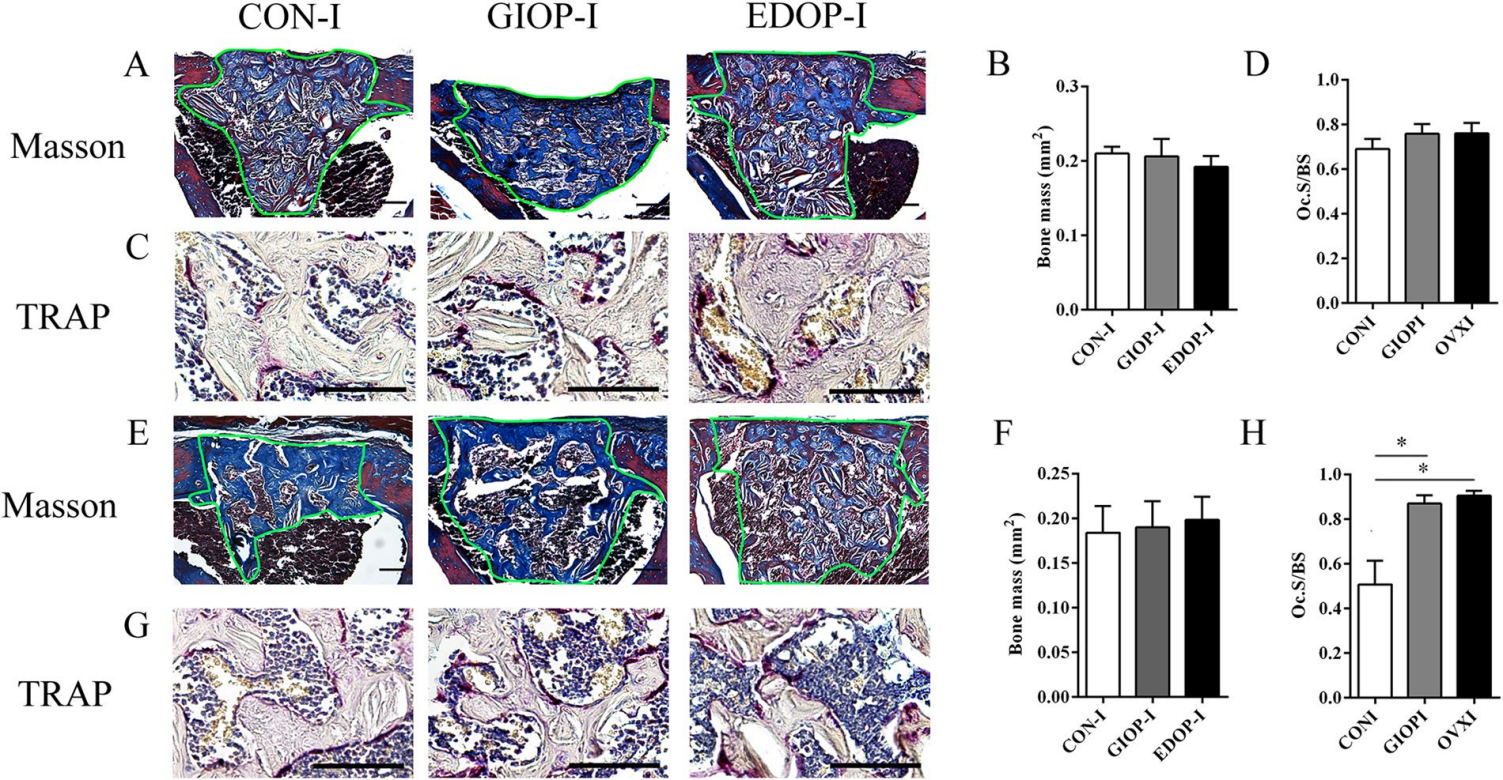
Histological and TRAP staining of the injury site 14 and 21 days after the drill-hole injury. (**A**) Callus sections 14 days after injury, stained with Masson’s trichrome. Scale bar, 100 µm. (**B**) Quantitative analyses of blue-stained bone mass, encircled by green lines. (**C**) TRAP staining of sections 14 days after injury. Scale bar, 50 µm (**D**) Quantitative analyses of TRAP + cells. (**E**) Callus sections 21 days after injury, stained with Masson’s trichrome. Scale bar, 100 µm. (**F**) Quantitative analyses of blue-stained bone mass, encircled by green lines. (**G**) TRAP staining of sections 14 days after injury. Scale bar, 50 µm (**H**) Quantitative analyses of TRAP+ cells. *P < 0.05.

Fourteen days after surgery, dense woven bone replaced the spicules woven bone in the callus area in group CON-I. The woven bone in the medullary area decreased compared with the level at 10 days after surgery, which indicated that the remodeling process had started (Fig. 3A). In groups GIOP-I and EDOP-I, the newly formed bone mass increased compared with the size at 10 days after surgery. No difference in the callus size was evident between these three groups (Fig. 3B). Tartrate-resistant acid phosphatase (TRAP) staining suggested that no difference in the osteoclast number per bone surface existed between the three groups (Fig. 3C,D).

Twenty-one days after surgery, the trabecular bone disappeared in the bone cavity in group CON-I. Instead, the lamellar cortical bone bridged the gap. A large amount of trabecular bone was still present in the bone cavities of groups GIOP-I and EDOP-I, suggesting that the remodeling process in both osteoporotic groups has been postponed (Fig. 3E,F). The number of TRAP-positive osteoclasts decreased in group CON-I; however, a large number were present in groups GIOP-I and EDOP-I (Fig. 3G,H), providing alternative evidence to support that the remodeling process in osteoporosis was delayed and defective.

### Chondrocyte mineralization and remodeling in endochondral ossification was delayed in both GIOP and EIOP

**Figure 4:**
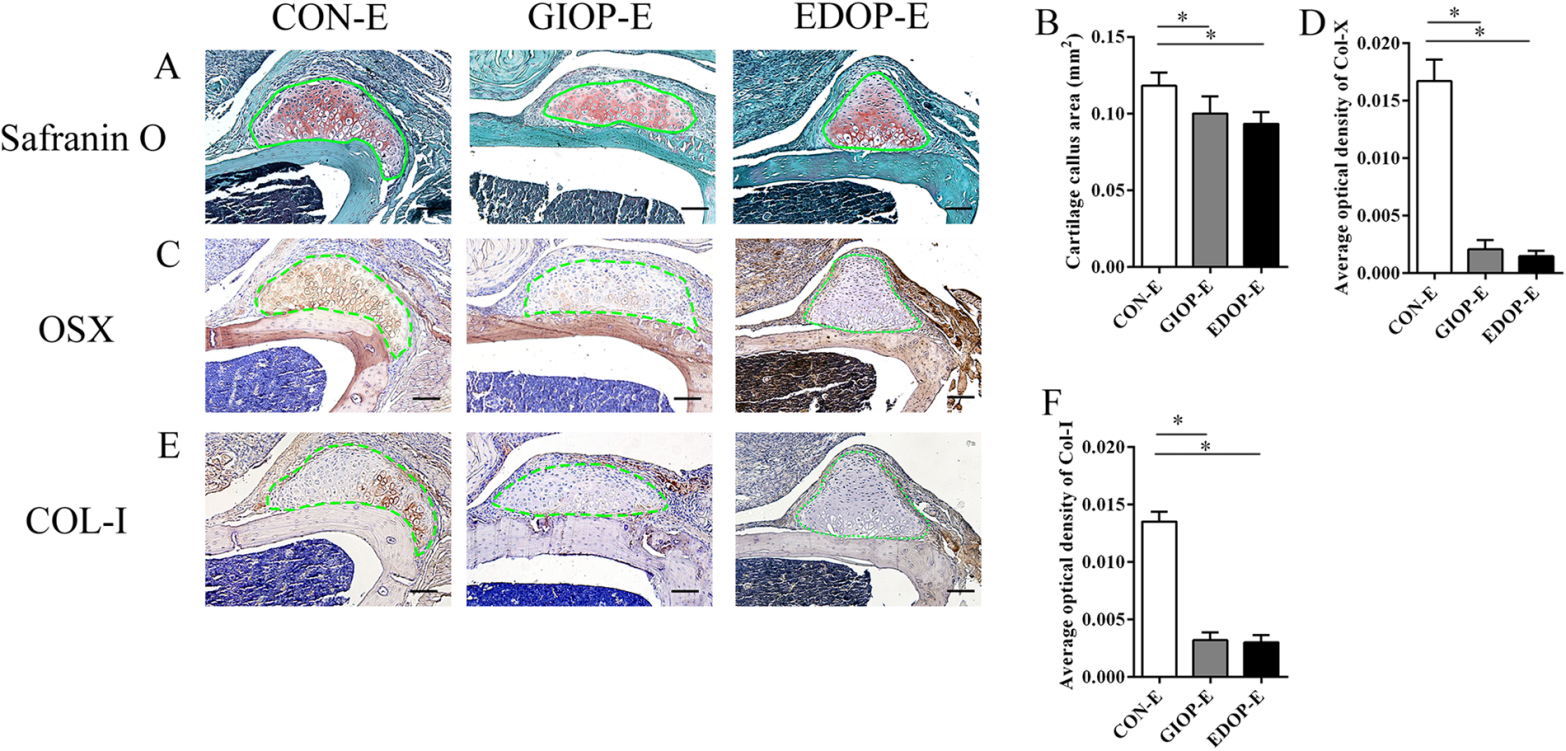
Analysis of the injury site 7 days after the periosteum injury. (**A**) Cartilage callus sections stained with Safranin O-Fast Green. (**B**) Analysis of the cartilage callus area. (**C**) Immunohistochemistry of type X collagen (COL-X) (brown). (**D**) Analysis of COL-X expressed in the callus areas. (**E**) Immunohistochemistry of type I collagen (COL-I) (brown). Scale bar, 100 µm. (**F**) Analysis of COL-I expressed in the callus areas. *P < 0.05.

Safranin O-Fast Green staining suggested that cartilage appeared in the scratched place 7 days after surgery (Fig. 4A). The cartilage callus was smaller in osteoporosis groups than in group CON-E (Fig. 4B). In group CON-E, many hypertrophic chondrocytes were present in the center of the cartilage callus. However, in groups GIOP-E and EDOP-E, only a few hypertrophic chondrocytes were evident (Fig. 4A). Collagen X (COL-X) is expressed specifically by mature hypertrophic chondrocytes^22,23^. The expression of COL-X was much stronger in the control group than in groups GIOP-E and EDOP-E, which exhibited small amounts of COL-X (Fig. 4C,D). Moreover, hypertrophic chondrocytes became osteoblasts during the process of endochondral ossification^24^. To evaluate whether ossification was also delayed, COL-I, a representative marker of osteoblasts, was used to evaluate mineralized cartilage^25^. COL-I was expressed in the mineralized cartilage around the hypertrophic chondrocytes in group CON-E. No COL-I was found in the cartilage areas in groups GIOP-E and EDOP-E (Fig. 4E,F). These data suggested that cartilage mineralization was defective in the osteoporosis groups.

**Figure 5:**
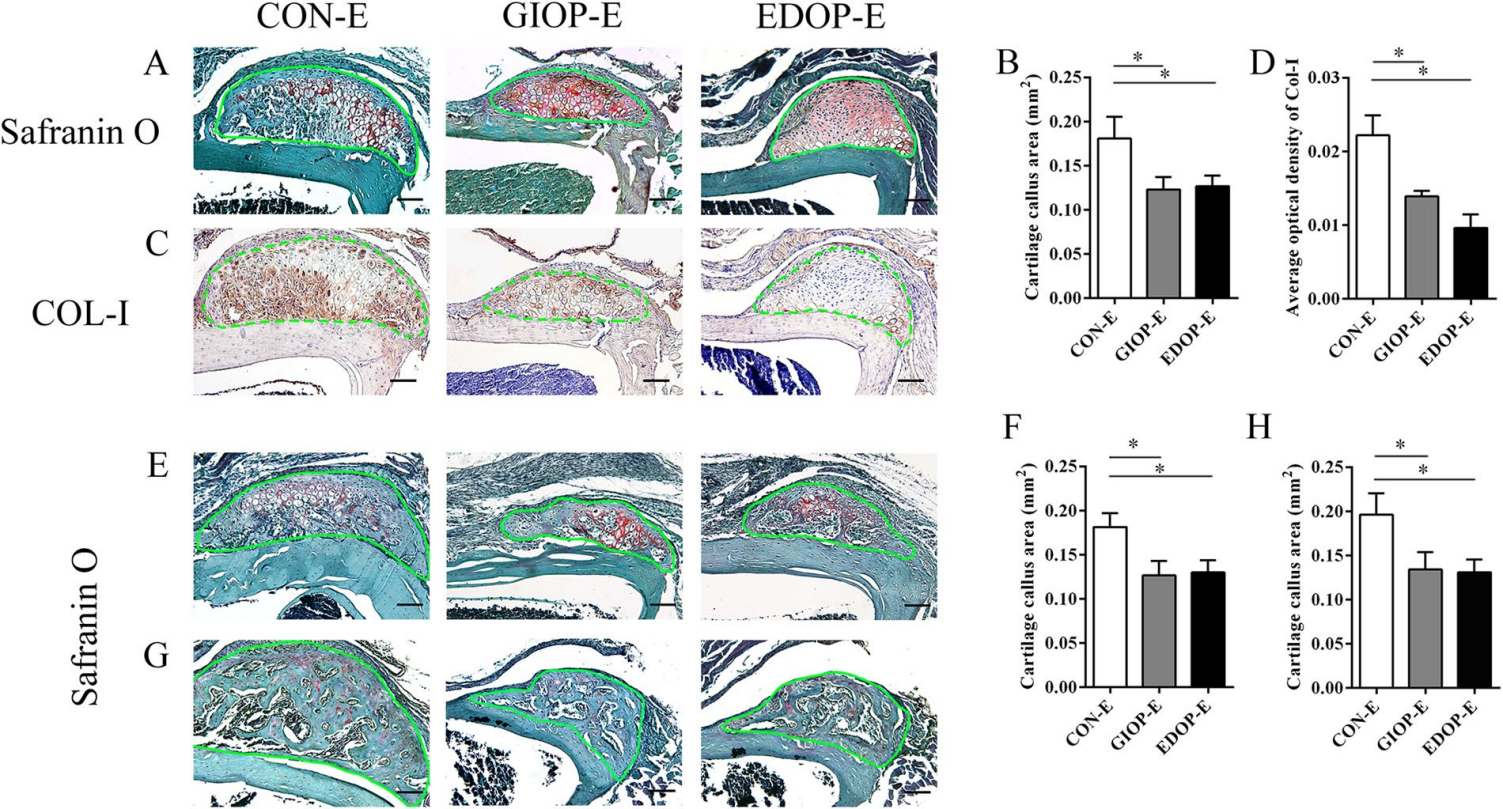
Analysis of the injury site at 10 days (**A**–**D**), 14 days (**E**,**F**) and 21 days (**G**,**H**) after the periosteum injury. (**A**) Cartilage callus sections stained with Safranin O-Fast Green. (**B**) Comparisons of the cartilage callus between the three groups. (**C**) Immunohistochemistry of type I collagen (COL-I) (brown). Scale bar, 100 µm. (**D**) Analyses of COL-I expression between the three groups. (**E**,**G**) Cartilage callus sections stained with Safranin O-Fast Green. (**F**,**H**) Callus area comparison on day 14 (**F**) and day 21 (**H**). *P < 0.05.

Ten days after surgery, the cartilage callus that had formed in the periosteum significantly expanded, but defective cartilage calluses were evident in both osteoporotic models (Fig. 5A,B). The trabecular bone appeared in the center of the cartilage matrix in group CON-E, indicating that ossification had started. Blood vessels also appeared to follow the ossification progression. In groups GIOP-E and EDOP-E, only the cartilage callus was observed (Fig. 5A). Further immunostaining analysis showed that COL-I was expressed robustly in the control cartilage callus (Fig. 5C,D).

Fourteen days after surgery, approximately one-third of the cartilage area was replaced by ossification and new trabecular bone in the control group. However, in the osteoporosis groups, cartilage mineralization and ossification had indeed started but with smaller calluses and less ossification (Fig. 5E). Similar to the concordant defective cartilage calluses in the osteoporosis groups on day 10 after surgery, the cartilage calluses on days 14 and 21 also showed smaller size compared with it in the control groups (Fig. 5E–G).

### Comparison of time-course callus formation area revealed the distinct impacts of GCs and ovariectomy on bone regeneration

**Figure 6:**
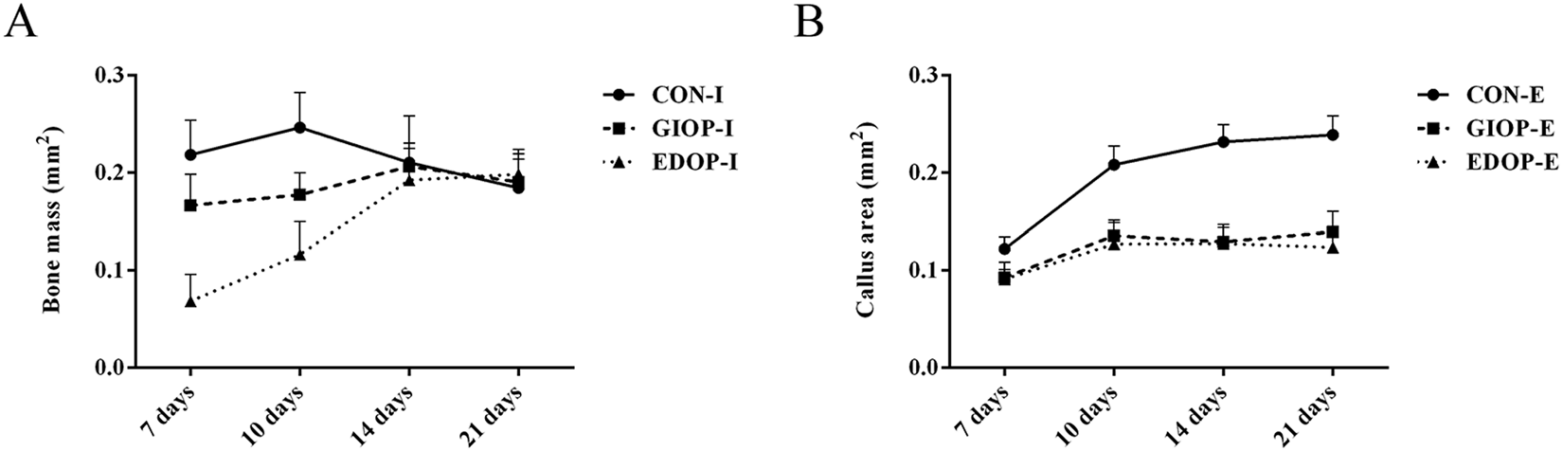
Change in the callus area from 7 days after surgery to 21 days after surgery. (**A**) The amount of newly formed bone mass change in intramembranous ossification. (**B**) Callus area change in endochondral ossification.

In the bone repair process via intramembranous ossification, the amount of newly formed bone mass in group CON-I reached its peak at 10 days after surgery and then quickly decreased until 21 days after surgery, when bone remodeling was almost complete. However, both the GIOP-I and EDOP-I groups showed defective and delayed callus formation (Fig. 6A). Osteoporosis affected not only the initial callus formation and callus peak but also the time to reach the peak callus, which was postponed to day 14~20 after surgery. Furthermore, group EDOP showed more obviously impaired regeneration than group GIOP, especially at the early stage. These data indicated that the initial regeneration through osteoprogenitor-mediated intramembranous ossification was affected more by estrogen deficiency.

Endochondral ossification occurred in the periosteum, the cartilage callus sizes of the three groups were almost similar at the beginning of cartilage formation at 7 days after surgery. Later, this periosteum-derived callus underwent robust cartilage expansion beginning on day 10. However, the osteoporotic cartilage callus did not expand accordingly, which might have been due to reduced chondrocyte hypertrophy, as it has been reported that chondrocyte hypertrophy largely responsible for about 60% of skeletal growth^26^. Histological analysis further demonstrated that cartilage mineralization and ossification were also impaired constantly (Fig. 6B).

### *In vitro* cell proliferation ability of BMSCs was impaired in GIOP and EDOP

**Figure 7:**
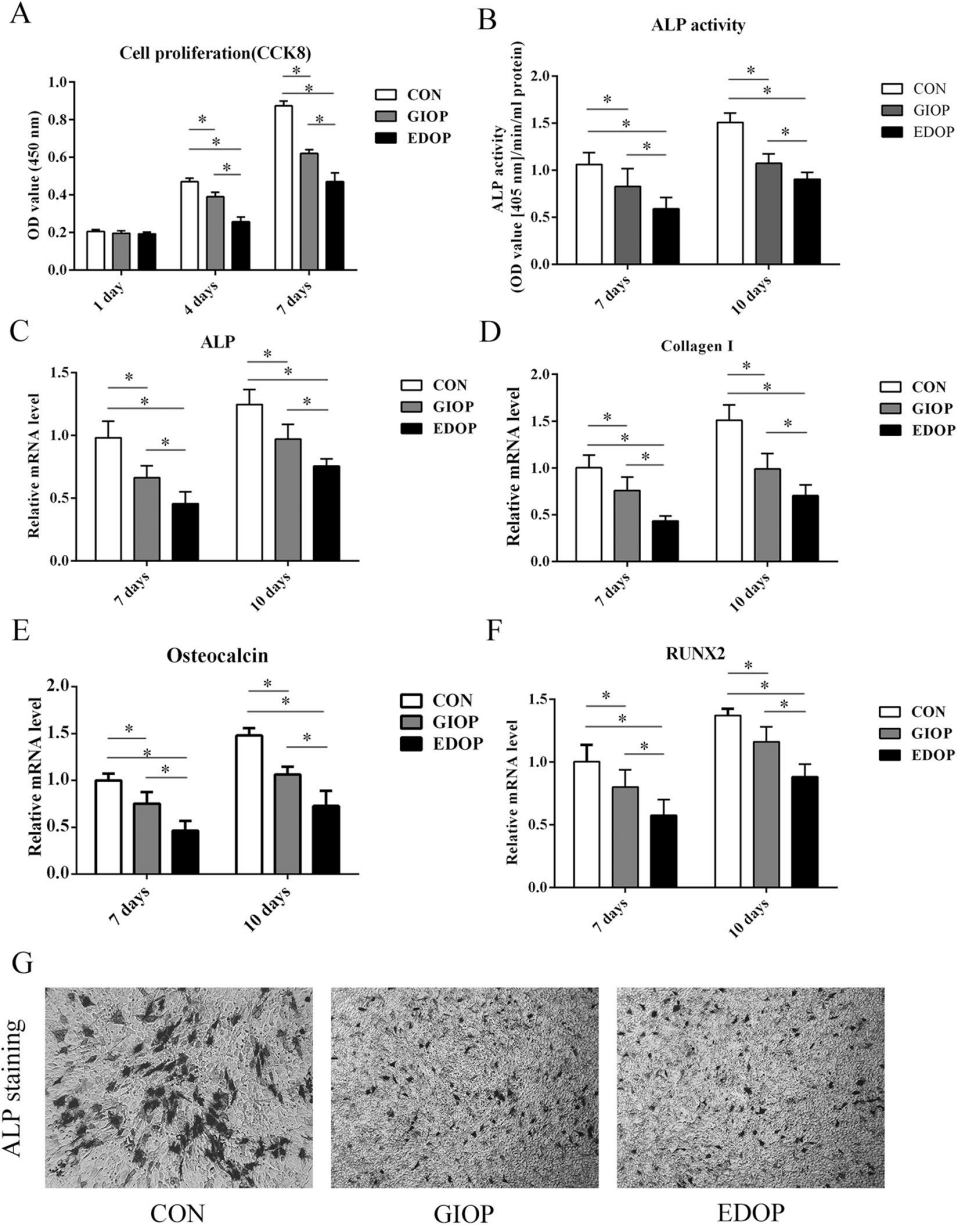
Quantitative analyses of the cell proliferation and differentiation of BMSCs. (**A**) Measurements of BMSC proliferation with the CCK-8 assay after 1, 4, and 7 days of incubation. (**B**) Analysis of alkaline phosphatase (ALP) activity at 7 and 10 days after incubation. mRNA expression levels were measured by RT-PCR: (**C**) ALP; (**D**) Collagen (**I**,**E**) Osteocalcin; (**F**) runt-related transcription factor 2 (RUNX2). (**G**) ALP staining of BMSCs at 14 days after incubation. *P < 0.05.

CCK-8 analysis was performed to evaluate the cell proliferation ability of BMSCs. No obvious difference was evident between the three groups after 1 day of incubation. Cell proliferation was significantly higher in group CON than in groups GIOP and EDOP at 4 and 7 days (P < 0.05). Moreover, cell proliferation was higher in group GIOP than in group EDOP (Fig. 7A). These results indicates that both GIOP and EDOP impaired BMSC proliferation and that proliferation was more seriously impaired in EDOP than in GIOP.

### *In vitro* osteogenic differentiation was impaired more seriously in EDOP than in GIOP

ALP is an important osteogenic marker of the early-stage osteogenesis differentiation of BMSCs^27^. ALP activity was measured on days 7 and 10 after incubation (Fig. 7B) and was lower in groups GIOP and EDOP than in group CON at both time points (P < 0.05). We also found that ALP activity was higher in group GIOP than in group EDOP (P < 0.05). The mRNA levels of multiple osteogenic differentiation markers (COL-I, osteocalcin [OCN], RUNX2) were quantified by real-time PCR (RT-PCR) to determine the expression levels of osteogenic genes. The results showed that the gene expression levels of COL-I, OCN and RUNX2 were significantly lower in groups GIOP and EDOP than in group CON (Fig. 7C–F). The gene expression levels were also lower in group EDOP than in group GIOP (P < 0.05). Moreover, ALP staining revealed lower osteoblast activity in groups GIOP and EDOP than in group CON (Fig. 7G). These results demonstrate that both GIOP and EDOP impair the osteogenic differentiation ability of BMSCs and that this ability is more seriously impaired in EDOP than in GIOP.

### The hypertrophy of chondrocytes in chondrogenic differentiation *in vitro* was impaired in EDOP and GIOP

**Figure 8:**
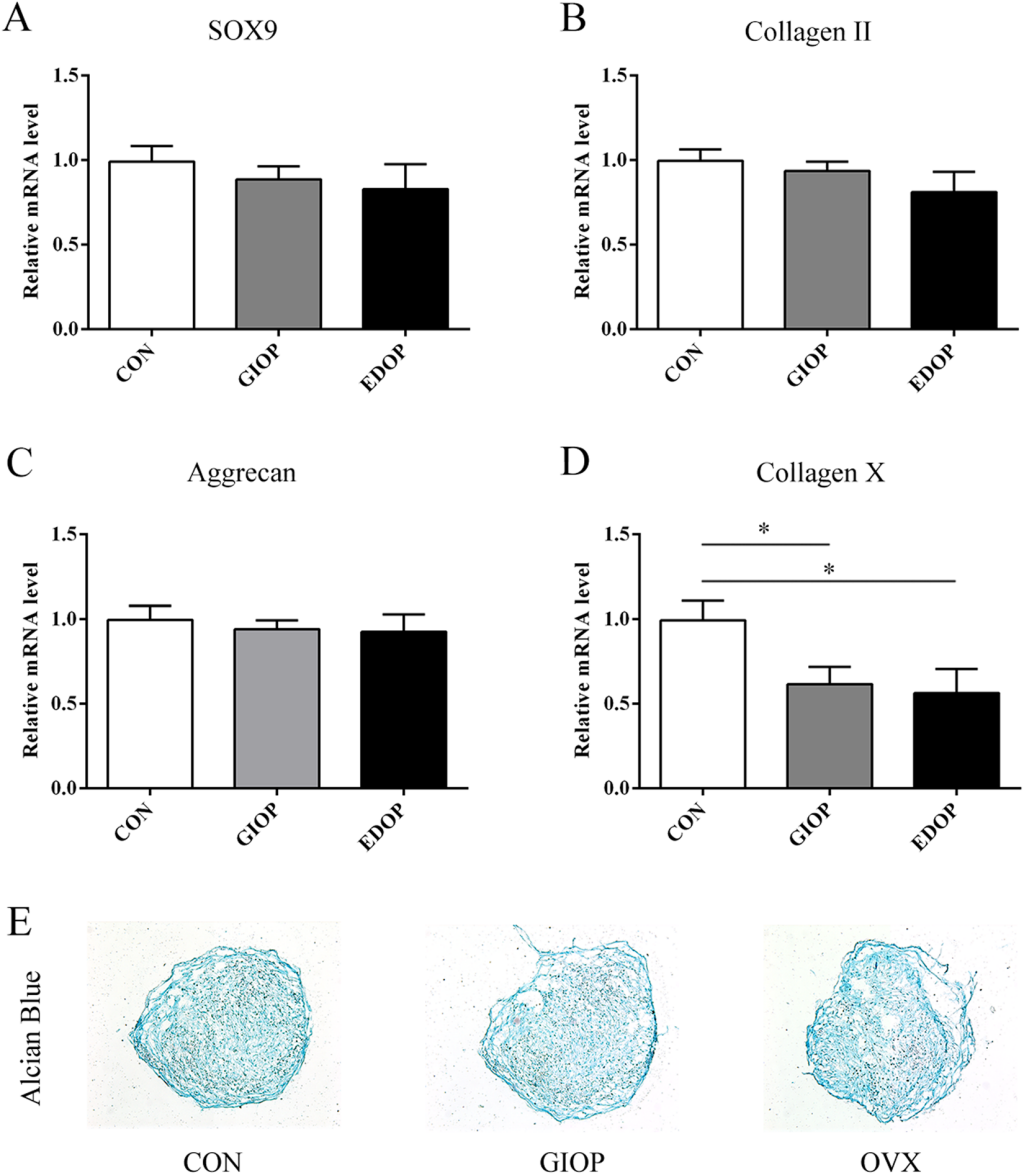
Effects of osteoporosis on chondrogenic differentiation and marker gene expression in bone mesenchymal stem cells (BMSCs). mRNA expression levels were measured by RT-PCR after 14 days of differentiation. (**A**) mRNA expression of sex-determining region Y-box 9 (SOX9); (**B**) mRNA expression of Collagen II; (**C**) mRNA expression of Aggrecan; (**D**) mRNA expression of Collagen X. (**E**) Alcian blue staining of chondrogenic pellets at 14 days after incubation. *P < 0.05.

Alcian blue staining of chondrogenic pellets suggested that BMSCs had become chondrocytes after 14 days of differentiation (Fig. 8E). The mRNA levels of collagen II (COL-II), COL-X, Aggrecan and SOX9 were quantified by RT-PCR to determine the chondrogenic differentiation ability of BMSCs 14 days after culturing in chondrogenic differentiation medium. The results showed that no differences were evident between the three groups in terms of the gene expression levels of COL-II, Aggrecan and SOX9; however, the COL-X expression level was significantly lower in groups GIOP and EDOP than in group CON (P < 0.05) (Fig. 8A–D). These results suggested that GIOP and EDOP mainly impaired the hypertrophy of chondrocytes in chondrogenic differentiation.

## Discussion

In the present study, we established two bone repair models that exhibited repair by intramembranous ossification or endochondral ossification. Additionally, this study is the first to show that intramembranous ossification and endochondral ossification are impaired differently between GIOP and EDOP. We found that EDOP delayed intramembranous ossification more seriously than GIOP, while both impaired endochondral ossification at the same level.

Fracture healing usually comprises intramembranous ossification and endochondral ossification^28^. Osteoporosis impairs fracture healing. However, whether intramembranous ossification and endochondral ossification are affected equivalently has remained unclear because no perfect fracture healing model has existed that could separate intramembranous ossification and endochondral ossification completely. The most common model of fracture healing is a break in the middle of the long bone^29–32^. This break is repaired by intramembranous ossification when the fracture is firmly fixed and repaired by both intramembranous ossification and endochondral ossification simultaneously when the fracture is not firmly fixed^33–36^. This model has many disadvantages; the model is expensive, difficult to manipulate, and limits the activity of the animal and the fixation device is easily causes inflammation. Most importantly, the mixing of intramembranous ossification and endochondral ossification makes a thorough understanding of the mechanism of repair during a bone fracture difficult. Recently, fracture models that involve drilling a hole in the middle of the femur or tibia, which is repaired by intramembranous ossification, have been reported^17,18,37^, and these models are very similar to our model; however, these reports have not established an endochondral ossification model, which means that existing models continue to focus on intramembranous ossification and ignore endochondral ossification. To our knowledge, the two bone repair models that we have established have not been reported. There models are advantageous for three reasons. First, these models are easier to manipulate and replicate than other fracture models. Second, these models are inexpensive because they do not require fixation devices. Third, the minimally invasive surgery reduces the suffering of the animal and reduces the incidence of inflammation. Fourth and most importantly, the models completely separate intramembranous ossification and endochondral ossification, enabling studies of how the fracture impacts the factors that influence each bone repair process.

**Figure 9:**
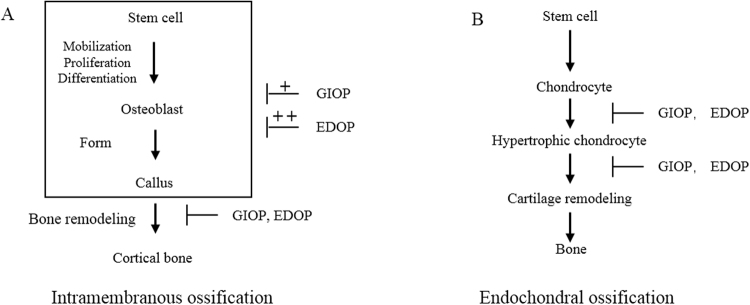
(**A**) General view of the influence of GIOP and EDOP on intramembranous ossification. (**B**) General view of the influence of GIOP and EDOP on endochondral ossification.

In our study, we also compared the different effects of GIOP and EDOP on these two bone repair models. In intramembranous ossification, the impact of GIOP on the bone remodeling process was more apparent than the of GIOP impact on BMSC proliferation and differentiation. However, the impact of EDOP on BMSC proliferation and differentiation was much more apparent than the impact of GIOP, while the effects of EDOP on the bone remodeling process were equivalent to those of GIOP (Fig. 9A). The hallmark of GIOP is a reduced bone formation rate^38^. Bone formation decreased dramatically over the first day of administration, and bone resorption transiently increased^39^. Glucocorticoids (GCs) impair osteoblast differentiation and proliferation by regulating Wnt signaling, BMP signaling and Notch signaling^40–42^. This regulation explains the impaired bone formation observed on days 7 and 10 after drill-hole injury in our study. In addition, a few studies have also suggested that excessive GCs induce osteoblast and osteocyte apoptosis^43,44^. Impaired bone formation and increased bone resorption rates results in a disordered bone remolding process in newly formed bone callus. This is not the case in EDOP. Bone resorption exceeds bone formation, but both are elevated in the pathology of EDOP^45^. The increased bone turnover is thought to be due in part to a shortening of the lifespan of osteoblasts and a prolongation of the lifespan of osteoclasts^46^. Consist with our study, Shi *et al*. reported that estrogen deficiency resulted in impaired osteogenesis^47^. As EDOP is a type of high-turnover osteoporosis, more BMSCs must be mobilized in EDOP than in to differentiate into osteoblasts to maintain a high bone formation rate compared with low-turnover osteoporosis. Kitajima, Y. *et al*. also reported that the number of bone marrow stem cells decreased in EDOP^48^. This may be the reason that the early phase of intramembranous ossification is badly impaired. Our study found that GCs and estrogen deficiency could differently affect the two repair processes. However, these processes need further extensive study in the future to fully understand their mechanism.

We are the first to report that in an endochondral ossification, using an endochondral ossification model, osteoporosis impairs endochondral ossification. We found that the difference in endochondral ossification was much smaller than the more distinct difference between GIOP and EDOP in terms of intramembranous ossification. In the beginning of endochondral ossification, the mobilization of BMSC differentiation to chondrocytes was slightly influenced, while the chondrocyte hypertrophy was clearly delayed. These phenomena were also apparent with intramembranous ossification, and the bone remodeling process was delayed in GIOP and EDOP (Fig. 9B). The results of the *in vitro* study were consistent with the *in vivo* conclusion. These results suggest that for different types of osteoporosis, different therapeutic strategies are needed to treat osteoporotic fractures.

In conclusion, our study successfully established a bone repair model that underwent repaired via intramembranous ossification or endochondral ossification and found that bone repair was differentially influenced by GIOP versus estrogen EDOP.

## Methods

### Experimental animals

One hundred thirty-five female BALB/c mice, aged 8 weeks, were obtained from the laboratory animal services center of The Fourth Military Medical University. The mice were housed for 2 weeks to acclimate to the environment before experimentation. The animal procedures were approved by The Fourth Military Medical University. All methods were performed in accordance with the relevant guidelines and regulations.

### Surgical protocol and experimental design

The mice were randomly divided into three groups: CON, GIOP, and EDOP. The mice in group CON were used as the blank control, the mice in group GIOP were subcutaneously injected with 3.5 mg/kg/day methylprednisolone for at least 8 weeks^49,50^, and the mice in group EDOP were subjected to ovariectomy. After 8 weeks, 5 mice from each group were euthanatized. The right femurs were collected for Micro-CT analysis to confirm the osteoporotic condition, and the left femurs were collected for *in vitro* cell culture. Then, each group was divided into two subgroups: the model of bone repair by intramembranous ossification (groups CON-I, GIOP-I and EDOP-I) and the model of bone repair by endochondral ossification (groups CON-E, GIOP-E, and EDOP-E).

The model of bone repair by IO was established with a drill-hole injury in the middle of anteromedial tibial. Mice were anesthetized by intraperitoneal injections of pelltobarbitalum natricum (80 mg/kg body weight). A skin incision was made at the middle of the left anteromedial tibia. Blunt dissection of the subcutaneous tissue was performed until the periosteum was exposed. A needle that was 0.7 mm in diameter was used to drill a hole into the anterior cortices. Afterward, the subcutaneous tissue was repositioned, and the skin was closed by suturing (Supplemental Fig. 2A).

The model of bone repair by EO was established with a scratch of the periosteum in the middle of the anteromedial tibial. After the mice were anesthetized and the periosteum was exposed, a needle tip was used to scratch the periosteum lengthwise in the middle of the left anteromedial tibia. The length of the scratch was 0.5–1 cm. Afterward, the subcutaneous tissue was repositioned, and the skin was closed by suturing (Supplemental Fig. 2B).

The mice from each group were sacrificed on days 7 (n = 5), 10 (n = 5), 14 (n = 5) and 21 (n = 5) after injury. 5-Bromo-2-deoxyuridine (BrdU) (MP, USA) (5 mg/kg) was injected intraperitoneally 2 hours before sacrifice on days 7 and 10.

### Micro-CT analysis of the distal femur

The bone microarchitecture of the distal femur was scanned using explore Locus SP Pre-Clinical Specimen microcomputed tomography (GE Healthcare, USA) with an 8-mm resolution, a tube voltage of 50 kV and a tube current of 0.1 mA. The reconstruction and 3D quantitative analysis were performed by using software provided by a desktop Micro-CT system (GE Healthcare, USA). The same conditions were used for all samples. The region of interest (ROI) was 0.5 mm away from the growth plate, the height was 2 mm, and the cortical region was excluded. The following 3D indices in the defined ROI were analyzed: BV/TV (%), Tb.N, Tb.Sp, trabecular thickness (Tb.Th), and BMD. The operator conducting the scan analysis was blinded to the treatments associated with the specimens.

### Histological examination

Samples were fixed in 4% paraformaldehyde for 48 hours and decalcified in 0.5 M Ethylene Diamine Tetraacetic Acid (EDTA). The samples were then embedded in paraffin. Sections (6 µm) were processed transversally along the tibial shaft axis and collected on glass slides. After deparaffinization, the slices were subjected to Masson’s trichrome staining using the manufacturer’s protocol (G1340; Solarbio, China), TRAP using the manufacturer’s protocol (Sigma #387 A; Sigma-Aldrich, USA), or Safranin O-Fast Green staining using the manufacturer’s protocol (G1371; Solarbio, China).

### Immunofluorescence

We use BrdU to mark proliferating cells and compare the cell proliferation rate between groups. After deparaffinization, the slices were rehydrated, washed with phosphate-buffered saline (PBS) and incubated in 1 N HCl on ice for 10 minutes. Then, the slices were incubated in 2 N HCl for 10 minutes at room temperature followed by 20 minutes at 37 °C. The slides were buffered in a 0.1 M sodium borate solution for 12 minutes at room temperature, washed with PBS, and blocked with 5% normal donkey serum (Millipore, USA) for 1 hour at room temperature. The slides were then incubated with a 1:50 dilution of a primary antibody, namely, sheep polyclonal anti-BrdU (ab1893, Abcam) at 4 °C overnight. Then, the slides washed with PBS and incubated with a secondary antibody, namely, donkey anti-sheep IgG conjugated to Cy3 fluorophores (Jackson ImmunoResearch, USA), diluted 1:500 in PBS, for 1 hour at room temperature. The slides were washed with PBS and incubated with DAPI for 10 minutes. After being washed with PBS, the slides were covered with coverslips, and examined by confocal microscopy.

### Immunohistochemistry

After deparaffinization, the slices were rehydrated; washed with PBS; incubated with 3% hydrogen peroxide at room temperature for 15 minutes; washed with PBS; incubated with hyaluronidase IV at 37 °C for 30 minutes; washed with PBS; blocked with 1:10 normal blocking serum at room temperature for 45 minutes; and incubated with a 1:100 dilution of anti-COL-I (ab84956, Abcam), a 1:100 dilution of anti-Osterix (ab22552, Abcam) or a 1:100 dilution of anti-COL-X (ab58632, Abcam) at 4 °C overnight. The slides were washed and incubated with a secondary antibody (sp9000, ZSGB-BIO) at 37 °C for 20 minutes, washed with PBS incubated with streptavidin peroxidase at 37 °C for 20 minutes. Then, the slides were washed with PBS and exposed for 5 minutes to the peroxidase DAB substrate (ZSGB-BIO, China). After staining with hematoxylin, the slides were examined with an Olympus microscope mounted with an Olympus video camera. We used Image-Pro Plus version 6.0 (Media Cybernetics, Inc.) to analyze the results. We first circled the area of interest and then measured the cumulative optical density (OD) of what we stained. Afterward, we determined the average OD with the cumulative OD divided by the size of the area of interest.

### Isolation and culture of bone marrow stem cells (BMSCs)

BMSCs were isolated from the left femurs and tibia of the mice sacrificed for Micro-CT analysis. Briefly, after the mice were subjected to cervical dislocation, BMSCs were obtained from the femurs and tibia by flushing the areas with low-glucose Dulbecco’s modified Eagle medium (DMEM; Thermo Fisher Scientific, Inc., Waltham, MA, USA) supplemented with 10% fetal bovine serum (FBS; Thermo Fisher Scientific, Inc.) and antibiotics (100 units/ml penicillin and 100 μg/ml streptomycin; Invitrogen, CA, USA). MSCs were seeded and incubated at 37 °C in 5% CO_2_. After 48 h, non-adherent cells were removed by changing the medium, and the medium was changed every 3 days thereafter. The remaining adherent cells were mainly BMSCs^51,52^. When the cells reached 80–90% confluence, they were trypsinized, counted, and reseeded. Cells from passages 3–5 were used for the experiments.

### Cell proliferation of BMSCs

The proliferation ability of BMSCs was evaluated using cell counting kit-8 (CCK-8, Japan). BMSCs were incubated in 24-well plates at a density of 15,000 cells/cm^2^. The proliferation rate of the BMSCs was estimated with CCK-8 assays on days 1, 4, and 7. During cultivation to these time points, the samples were transferred to new 24-well culture plates. CCK-8 solution with a 10% volume of the medium was then added to the wells, and the samples were incubated at 37° for 2 h. Afterward, 100 μl the reaction solution was transferred to a new 96-well plate, and the OD was measured at 450 nm with a microplate reader.

### Osteogenic differentiation

BMSCs were seeded at a density of 15,000 cells/cm^2^ in 6-well plates for quantitative RT-PCR and 12-well plates for the alkaline phosphatase (ALP) activity assay in DMEM with 10% FBS^53^. When the cells reached 70–80% confluence, the growth medium was changed to osteogenic differentiation medium (Cyagen Biosciences Inc, China). For the ALP activity and RT-PCR assays, cells were treated for 7 and 10 days with osteogenic differentiation medium. For ALP staining, cells were treated for 14 days with osteogenic differentiation medium.

### Chondrogenic differentiation

BMSCs were seeded at a density of 10,000 cell/cm^2^ in 6-well plates for RT-PCR. When the cells reached 70–80% confluence, the growth medium was changed to chondrogenic differentiation medium (GUXMX-90041, Cyagen Biosciences Inc, China). The medium was changed every 3 days, and induced cartilage tissues were harvested on day 14 for RT-PCR analysis. We performed the chondrogenic pellet cultures according to the manufacturer’s protocol. The chondrogenic pellets were then formalin-fixed and paraffin-embedded for Alcian blue stain analysis.

### Alkaline phosphatase (ALP) activity assay

The ALP activity was measured with the ALP assay at 7 and 10 days after treatment with osteogenic differentiation medium. The assays were performed as previously described^54^. The ALP activity was expressed as the OD value per the total protein amount, which was determined using BCA protein assay kits and a series of BSA standards.

### Quantitative real-time PCR

The expression levels of osteogenic marker genes, namely, ALP, COL-I, OCN, and runt-related transcription factor 2 (RUNX2), and chondrogenic marker genes, namely, COL-II, COL-X, aggrecan, and sex-determining region Y-box 9 (SOX9), were examined. Briefly, total RNA was extracted from cells using the RNAiso Plus reagent (Takara Biotechnology Co., China) and then converted to cDNA using the Prime Script RT master mix (Takara Biotechnology Co., Ltd.), according to the manufacturer’s protocols. The RT-PCR reactions were performed using SYBR Premix Ex Taq II (Takara) on a PCR System (Bio-Rad). The RNA concentration was determined by measuring the optical absorbance of the extract at 260 nm. The housekeeping gene, GAPDH, was used as an internal control. The primer sequences are shown in Table 1.

**Table 1:** Primer sequences for the real-time PCR analysis of gene expression.

Target gene	Forward PCR primer (5′-3′)	Reverse PCR primer (5′-3′)
ALP	GCAAGGACATCGCATATCAGC	GGCCTTCTCATCCAGTTCGTA
Collagen I	GAGCGGAGAGTACTGGATCG	GCTTCTTTTCCTTGGGGTTC
Osteocalcin	ACCATCTTTCTGCTCACTCTGCT	CCTTATTGCCCTCCTGCTTG
RUNX2	TGGCAGCACGCTATTAAATC	TCTGCCGCTAGAATTCAAAA
Collagen II	CACACTGGTAAGTGGGGCAAGACCG	GGATTGTGTTGTTTCAGGGTTCGGG
Collagen X	CAAACGGCCTCTACTCCTCTGA	CGATGGAATTGGGTGGAAAG
Aggrecan	AGGGTGAGAAGGTAAGGGGT	GAGGCGAAGTAACCAACCAT
SOX9	TGAATCTCCTGGACCCCTTC	TGCTGGAGCCGTTGACGCG
GAPDH	TGCTGGTGCTGAGTATGTGGT	AGTCTTCTGGGTGGCAGTGAT

### Statistical analysis

Statistical analyses were performed using SPSS software, version 15.0 (SPSS Inc., USA). Quantitative data are presented as the mean ± standard deviation and were compared using one-way analysis of variance with post hoc analysis to determine the significance among the different groups.

### Data Availability

The datasets generated during and/or analysed during the current study are available from the corresponding author on reasonable request.

## Electronic supplementary material

Supplement Figures
