# Supplementary material for: Intramembranous ossification and endochondral ossification are impaired differently between glucocorticoid-induced osteoporosis and estrogen deficiency-induced osteoporosis
Source: Sci Rep. 2018 Mar 1;8:3867. doi: 10.1038/s41598-018-22095-1 (PMC5832871; doi:10.1038/s41598-018-22095-1)
Supplement: Supplementary file 1 — Supplement Figures [file 41598_2018_22095_MOESM1_ESM.docx]

**Intramembranous ossification and endochondral ossification are impaired differently between** **glucocorticoid induced osteoporosis and** **estrogen deficiency induced osteoporosis**

Hongyang Zhang^1, +^, Xiaojuan Shi ^1, +^, Long Wang ^2, +^, Xiaojie Li ^3, +^, Chao Zheng^1^, Bo Gao^1^, Xiaolong Xu^1^, Xisheng Lin^1^, Jinpeng Wang^1^, Yangjing Lin^4^, Jun Shi^5^, Qiang Huang^6^, Zhuojing Luo^1, *^, Liu Yang^1, *^

^1^Institute of Orthopedic Surgery, Xijing Hospital, The Fourth Military Medical University, Xi’an 710032, People’s Republic of China.

^2^Department of Orthopaedics, Chinese PLA General Hospital, Beijing, 100853, People's Republic of China.

^3^Department of Orthopeadics, Air Force General Hospital, Beijing 100142, People's Republic of China.

^4^Department of Orthopaedics, First Affiliated Hospital, Chengdu Medical College, No.278, Baoguang Road, Chengdu, SiChuan Province 610500, People’s Republic of China.

^5^Department of Orthopedics, No.371 Central Hospital of PLA, Xinxiang, Henan 453000, People’s Republic of China.

^6^Lanzhou General hospital of Lanzhou Military Command, Lanzhou Gansu, 730050, People’s Republic of China.

***Corresponding authors:**

Liu Yang, Ph.D., Institute of Orthopedic Surgery, Xijing Hospital, The Fourth Military Medical University, Xi’an, People’s Republic of China. E-mail: [yangliu@fmmu.edu.cn](mailto:yangliu@fmmu.edu.cn). Tel: 86-29-84775291

Zhuojing Luo, Ph.D., Institute of Orthopedic Surgery, Xijing Hospital, The Fourth Military Medical University, Xi’an, People’s Republic of China. E-mail: zjluofmmu@hotmail.com. <Tel:86-29-84775275>

+these authors contributed equally to this work


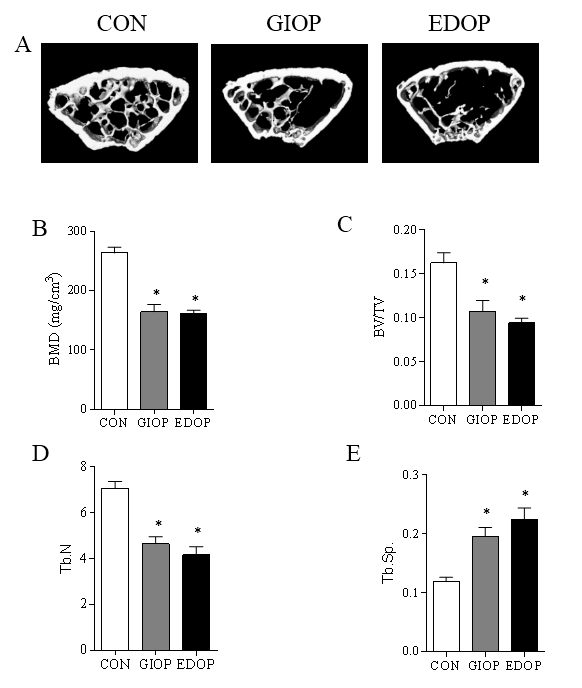


**Supplemental Figure 1.** Comparison of bone mass between three groups. A: Transverse plane of distal femur scanned by MicroCT. B-D: Statistical analysis of bone quality by MicroCT. CON: blank control; GIOP: 8 weeks after subcutaneously given 3.5 mg/kg/day methylprednisolone; OVX: 8 weeks after ovariectomy. *:p<0.05


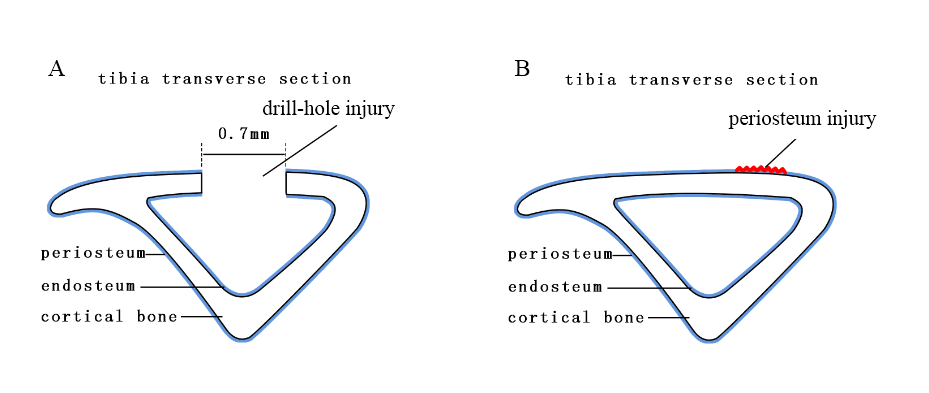


**Supplemental Figure 2.** Schematic diagram of bone repair model. A: Schematic diagram of intramembranous ossification. B: Schematic diagram of endochondral ossification.
